# Comparative Snake Venom Analysis for Facilitating Wildlife Forensics: A Pilot Study

**DOI:** 10.1155/2022/8644993

**Published:** 2022-06-03

**Authors:** Saurabh Bhargava, Kiran Kumari, Rajendra Kumar Sarin, Rajvinder Singh

**Affiliations:** ^1^Department of Forensic Science, Maharshi Dayanand University, Rohtak 124001, Haryana, India; ^2^School of Advanced Sciences & Languages, VIT Bhopal University, Bhopal, Madhya Pradesh, India; ^3^Forensic Science Department, Lovely Professional University, Phagwara (144001), Punjab, India; ^4^National Forensic Science University, Goa Campus, India

## Abstract

Confirm and authentic identification of species is required for the implementation of wildlife laws in cases of illegal trafficking of snake venoms. Illegally trafficked snake venom might be misidentified with other drugs of abuse, and sometimes, the species of venom-yielding snake cannot be verified. Snake venoms from medically important snake species, *Naja naja* and *Daboia russelii,* were procured from Irula Snake Catcher's Society, Tamil Nadu, India. Comparative analyses of both venoms were carried out using SDS-PAGE, LC-MS/MS, ICP-MS, and mtDNA analysis. The protein concentration of *Naja naja* and *Daboia russelii* venoms was 76.1% and 83.9%, respectively. SDS analysis showed a distinct banding pattern of both venoms. LC-MS/MS results showed proteins and toxins from 12 to 14 protein families in *Naja naja* and *Daboia russelii* venoms. Elemental analysis using ICP-MS showed a different profile of some elements in both venoms. mtDNA analysis of venoms using universal primers against *Cyt b* gene showed homology with sequence of *Naja naja* and *Daboia russelii* genes. The study proposed a template of various conventional and advanced molecular and instrumental techniques with their pros and cons. The template can be used by forensic science laboratories for detection, screening, and confirmatory analysis of suspected venoms of snakes. Clubbing of various techniques can be used to confirm the identification of species of snake from which the alleged venom was milked. The results can be helpful in framing charge-sheets against accused of illegal venom trafficking and can also be used to verify the purity and quality of commercially available snake venoms.

## 1. Introduction

Snakes and their venoms are illegally trafficked across the world for various purposes including medical and nonmedical uses. However, there is dearth of exact data on such illegal trafficking, but cases reported by TRAFFIC and World Wide Fund for Nature (WWF) have clearly shown the magnitude of such illegal trafficking of snakes and their venoms [1–3]. *Naja naja* and *Daboia russelii* are medically most relevant snake species in Indian subcontinent causing maximum fatality. In addition, these two species are the most traded venomous snake species across the world [4]. International trade of *Naja naja* and *Daboia russelii* and their products is regulated under Convention on International Trade in Endangered Species (CITES), where these are placed in APPENDIX II and III, respectively (https://www.traffic.org/; https://speciesplus.net/). Both these species are also regulated by the Wildlife (Protection) Act, 1972 of India and are placed in Schedule II (Part II) of endangered species (https://wiienvis.nic.in/Database/ScheduleSpecies%20Database_7969.aspx). However, the effective implementations of such legislations have remained a challenge to authorities because of several factors. One such factor is the limitation of forensic science in ascertaining the origin of such seized samples that lack physical markers of the identification of animal species. Snake venoms belong to this category of seizures by law enforcement authorities around the world, which, in the absence of physical markers, are required to be associated with a particular species to frame the charges against the accused.

The proteinaceous nature of snake venoms has been established since the 19th century. The exact composition of each snake species varies considerably. The venom of each venomous snake is a highly complicated cocktail of up to hundreds of enzymes, peptides, and toxins among other components [5, 6]. The recent advancements in scientific research have helped identify, purify, and isolate various bioactive compounds from snake venoms, which have great potential to be used in/as medicines. These potential pharmaceuticals extracted from venoms are not limited to the treatment of snake envenomations, but some bioactive chemicals have shown promising uses in the management of various diseases related to blood, brain, cancer, analgesic class, etc [7–17].

The venoms have also been reported to be used as a substance of abuse by creeps as alternatives to neurotic stimulants [18, 19]. The versatile uses of snake venoms have caused a higher demand of snake venoms, which further caused manifold increase in the price of crude snake venoms. The price of crude venom keeps on increasing because of many reasons, which include lesser yield per milking, lesser number of snakes due to excessive killing/habitat encroachment, restrictions by wildlife laws of different territories, and dangerous process of milking of venom. The price in illegal market has skyrocketed, which again caused the excessive illegal trafficking of venoms worth millions of dollars every year, thereby posing a great deal of threat to the existence of some snake species.

Wildlife authorities and other law enforcement agencies quite often seize illegally procured venoms of endangered species when trafficked across state/country borders (https://www.wwfindia.org/about_wwf/enablers/traffic/buzz.cfm). The accused are charged with wildlife laws of concerned states. However, on many occasions, the states' attorneys fail to prove that the seized material to be venom, thereby resulting in acquittal of accused **(**https://www.casemine.com/judgement/in/56ea9506607dba38b6e49b5a**)**. Similar identification of venom-yielding snake species is also required in the verification of commercially available pooled venoms. In the absence of physical markers, the venom cannot be confirmed unless tested in a forensic laboratory. A very dismal state of forensic analysis of snake venoms has been established [20].

However, the venoms have been studied using immunological, molecular, and instrumental techniques [21], but the use of these techniques for solving wildlife-related crimes of snake venom trafficking is scarce. Clubbing of a preliminary screening of venom and a more sophisticated venom profiling technique can be used in establishing the uniqueness of venoms for forensic purposes. A comprehensive knowledge about venom composition can help in establishing the distinguished identity of each venom. Therefore, a comprehensive compendium of scientific analysis for the identification of the venom sample has been devised with the help of this imperative piece of research. In this study, we have compared the profiles of venoms of the two most commonly encountered and medically relevant snake species of India: *Naja naja* and *Daboia russelii*. Various techniques, with their pros and cons in forensic venom analyses are described. The outcome of this research work is likely to help forensic experts in snake venom analysis, and will definitely help in better implementation of wildlife protection laws and aid-in conservation of endangered snake species.

## 2. Materials and Methods

### 2.1. Snake Venom Samples

**Figure 1:**
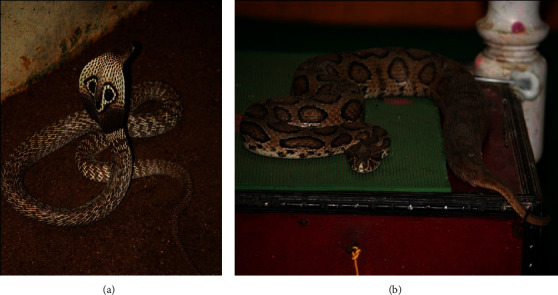
(a) Indian cobra *(Naja naja)* and (b) Russell's viper *(Daboia russelii)* (pictures were captured by one of the authors on 21 March 2018).

One Gram lyophilized crude venom of each *Naja naja* (Figure 1(a)) and *Daboia russelii* (Figure 1(b)) snakes was commercially procured purely for research purpose from “The Irula Snake Catchers Industrial Cooperative Society Limited, Tamil Nadu (India)” (vide Invoice No. 024 Dated 11.03.18). These samples were purchased after obtaining mandatory permissions from Haryana State Forest Department [Letter No. 5460 dated 09-Dec-2015], Institutional Animal Ethical Committee (IAEC) [Letter No. 360–73 dated 05-May-2016], and Institutional Bio-safety Committee (IBSC) [Letter dated 30-May-2017]. Venom samples were kept at -20°C storing condition in the toxicology laboratory of the department before use and later handled as per the standard operating procedure dictated by IBSC during the research analysis.

### 2.2. Ouchterlony Diffusion Analysis

Double diffusion Ouchterlony method was used as per the standard protocol [22]. Agarose (0.8%, 15 ml) was poured in a clean and sterile Petri dish and allowed to solidify. Wells were punched out in circular pattern around a central well. Thirty microtitre (30 *µ*l) of polyvalent snake venom antiserum against “*Big Four”* (Batch no. A05318001; Bharat Serums and Vaccines Limited, India) was poured in the central well. Venom of *Naja naja* and *Daboia russelii* (20µl each), human saliva (positive control), and distilled water (negative control) were poured in the surrounding wells. The Petri dish was kept in a sterile environment at room temperature for antigens and antibody precipitation reaction. The presence of precipitin band in gel matrix was observed from 24 to 72 hours.

### 2.3. Protein Quantification

Protein quantification was performed using the standard bovine serum albumin (BSA) method [23]. Coomassie Blue B G-250 (CBB G-250), phosphoric acid, ethanol, and bovine serum albumin (BSA) were purchased from Sigma-Aldrich. One milligram snake venom sample of each cobra and viper was dissolved in 1 ml distilled water in different vials. The protein concentration in this stock solution (1 mg/ml) was estimated using the Bradford method as described in the literature. A standard curve was plotted against known BSA concentrations and absorbance values. The concentration of proteins in our study samples were deduced using the equation derived from the standard curve.

### 2.4. mtDNA Analysis

DNA extraction solution (Cat no. 612104680501730, GeNei Laboratories Pvt Ltd (GeNei™)) isolated DNA for subsequent sequencing of the mitochondrial gene, from 10 mg venom sample of each snake. The extracted DNA samples were electrophoretically quantified using 0.8% agarose gel. A gel documentation system (DNR Bio-Imaging Systems) was used for photographic records of results. Isolated DNA samples were kept at -20°C for PCR amplification. Mitochondrial cytochrome b *(cyt b)* gene was amplified using universal primers set mcb 398 (5′TACCATGAGGACAAATATCATTCTG3′) and mcb 869 (3′CCTCCTAGTTTGTTAGGGATTGATCG5′) [3,24]. PCR amplification reaction was performed in a 25 *μ*l volume of the reaction mixture, 1X of PCR buffer (10 mM Tris-HCl, pH 8.3, and 50 mm KCl), 1.0 *µ*l of 2.5 mM each of dNTP mix, 3U*Taq* polymerase, 20pmol of each primer, 20 ng genomic template DNA, and distilled water to make the final volume. PCR thermal reaction consisted of an initial denaturation at 94°C for 5 min; followed by 35 cycles at 94°C for 30 seconds, 49°C for 45 seconds, and 72°C for 45 seconds; and a final extension at 72°C for 7 minutes. PCR-amplified products of related band size for both, mcb 400 bp along with StepUp^TM^ 100 bp DNA ladder (Cat# 2651970501730, Genei (GeNei™) Laboratories Pvt Ltd) were checked on 2% agarose gel. The nucleotide sequencing was performed using Sanger's dideoxy method. The sequencing was outsourced to GeNei Laboratories Pvt. Ltd, Bangalore, India. The forward sequences were aligned on NCBI blast **(**https://blast.ncbi.nlm.nih.gov/Blast.cgi**)** to match query sequence percentage from the nucleotide database.

### 2.5. SDS-PAGE

Using the Laemmli method [25], 12% SDS-PAGE was run with 2 mg/ml concentration of venom samples. Samples at a final concentration of 2 mg/ml were prepared in sample buffer containing 15 mM DTT. Twenty microliter (20 *µ*l) of both *Naja naja* and *Daboia russelii* venom samples were electrophoresed in different lanes at 180 V for 40 minutes on SDS-PAGE. Broad Range (3.5–205 kDa) protein molecular weight marker (GeNei) was used as marker protein in one lane of gel. Gels were then stained overnight with 0.1% CBB R-250 and distained with 10% acetic acid. Gel was photographed using Bio Imager (Alpha Inotech, San Leandro, California, USA). The banding pattern was qualitatively evaluated and compared for both venom samples.

### 2.6. Liquid Chromatography Tandem Mass Spectrometry (LC-MS/MS)

Untargeted qualitative proteomic profiling of whole venoms of *Naja naja* and *Daboia russelii* was performed using LC-MS Orbitrap. Sample was incubated with 6 M GdnHCl (Sigma) in 25 mM ammonium bicarbonate (pH∼7) overnight at 37^0^ C and then reduced with 10 mM DTT (Sigma) in LC-MS grade water (pH∼7) at 55^0^ C for 45 minutes. Samples were alkylated using 55 mM IAA (Sigma) in 25 mM ammonium bicarbonate (pH∼7) at room temperature in dark for 30 minutes. The samples were diluted to 1 M GdnHcl by 25 mM ammonium bicarbonate. Trypsin (Promega) was added in a ratio of 1 : 50 for overnight digestion at 37^0^ C. Desalting of samples was carried out as per the manufacturer's protocol (https://assets.thermofisher.com/TFS-Assets/LSG/manuals/MAN0011495_Pierce_C18_SpinCol_UG.pdf). The samples were speed vac till dry and finally reconstituted in 0.1% formic acid. 3*µ*g was loaded on C18 reverse-phase column. Peptides were separated by nano-LC (Thermo Scientific Easy-nLC 1200), which was connected to Thermo Scientific Q Exactive Orbitrap mass spectrometer. PepMap RSLC C18 2 *µ*m × 50 cm (Thermo scientific) column was used for elution. Injection volume was adjusted to 10 *μ*l per sample, using a flow rate of 0.3 *μ*l/min, with a linear gradient of 5–95% of solvent B (0.1% formic acid in 95% acetonitrile). Various machine and experiment parameters of MS/MS analysis are tabulated in Table 1. Raw data were obtained and analyzed against *Serpentes* database of Swiss-Prot and TrEMBL of UniProt database (https://www.uniprot.org/) using Proteome Discoverer 2.2 (Thermo Scientific). The LC-MS/MS experiment was conducted at Central Instrument Facility of Delhi University (South Campus), New Delhi.

### 2.7. Elemental Analysis with ICP-MS

Open acid digestion method was devised for venom sample preparation for ICP-MS. Modification of the digestion method described by Tarantino et al. [26] was used for the digestion of organic matter. Both venom samples (190 mg each) were digested using *aqua regia* (ICP grade pro-analytical acids from Merck were used). The tedious procedure of digestion of sample using *aqua regia* involved dropwise addition of 2 ml of H_2_O_2_ to 190 mg of venom samples in crucibles followed by heating at 60°C till effervescence (80 minutes). Then, 10 ml *aqua regia* was added dropwise and heated until it evaporated completely. 20 ml 2N HNO_3_ was added to bring the digested sample in solution form. Solution was swirled and transferred to volumetric flask. The flask was gently heated to dissolve crystalline matter. The clear solution was then cooled, and a final volume of 25 ml was made with Milli-Q water. The Agilent 7900 ICP-MS was used for the elemental analysis of digested samples of both snake venoms. Nebulizer gas flow was ∼1 L/min, auxilliary gas flow was ∼1 L/min, plasma gas flow was ∼15 L/min, and helium (He) gas flow in the reaction cell was kept at ∼0.2 mL/min. Reflected power of ∼45 W and forward power of ∼1500 W were maintained. Analyzer vacuum was maintained at ∼6 × 10^−5^. 5% HNO_3_ solution (Merck) was used as blank.

Standard solutions (A and B) were prepared as STD A containing 33 elements = 10 ppm solution (Sigma-Aldrich = Pcode: 101529525 = 92091–100 ML) and STD B containing 17 elements = 10 ppm solution (Sigma-Aldrich = Pcode: 101573448 = 41135–100 ML). Trace metal elemental analysis of both samples was carried out for four S-block elements (sodium, potassium, magnesium, and calcium), eight D-block elements (vanadium, chromium, manganese, iron, cobalt, nickel, copper, zinc), and one P-block element (aluminum). The results were acquired in ppb (parts per billion) concentrations and later converted to ppm (parts per million).

## 3. Results

### 3.1. Ouchterlony (Immuno-Diffusion)

**Figure 2:**
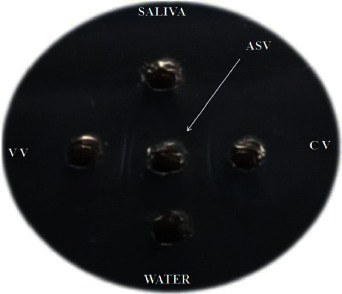
Immunodiffusion testing of venoms of *Naja naja* and *Daboia russelii* with commercially available polyvalent antiserum against Big Four. VV: viper venom (left well); CV: cobra venom (right well); ASV: antisnake venom (center well).

Distinct precipitin bands appeared between wells containing venom samples and well containing antiserum (Figure 2). Precipitin band between the venom of *Daboia russelii* and antiserum was more prominent and started appearing earlier at around 24 hrs, while precipitin band between the venom of *Naja naja* and antiserum was less prominent and started appearing at 36 hours. However, there was not any precipitin band between wells containing human saliva and water taken as control samples with antiserum well. The results tentatively detected snake venom taken as research samples.

### 3.2. mtDNA Analysis

The electrophoretically extracted DNA using 0.8% agarose gel documentation system and PCR-amplified products are shown in Supplementary Figure 1. The nucleotide sequences of mtDNA were determined for *Naja naja* and *Daboia russelii* snake species in this study.

**Figure 3:**
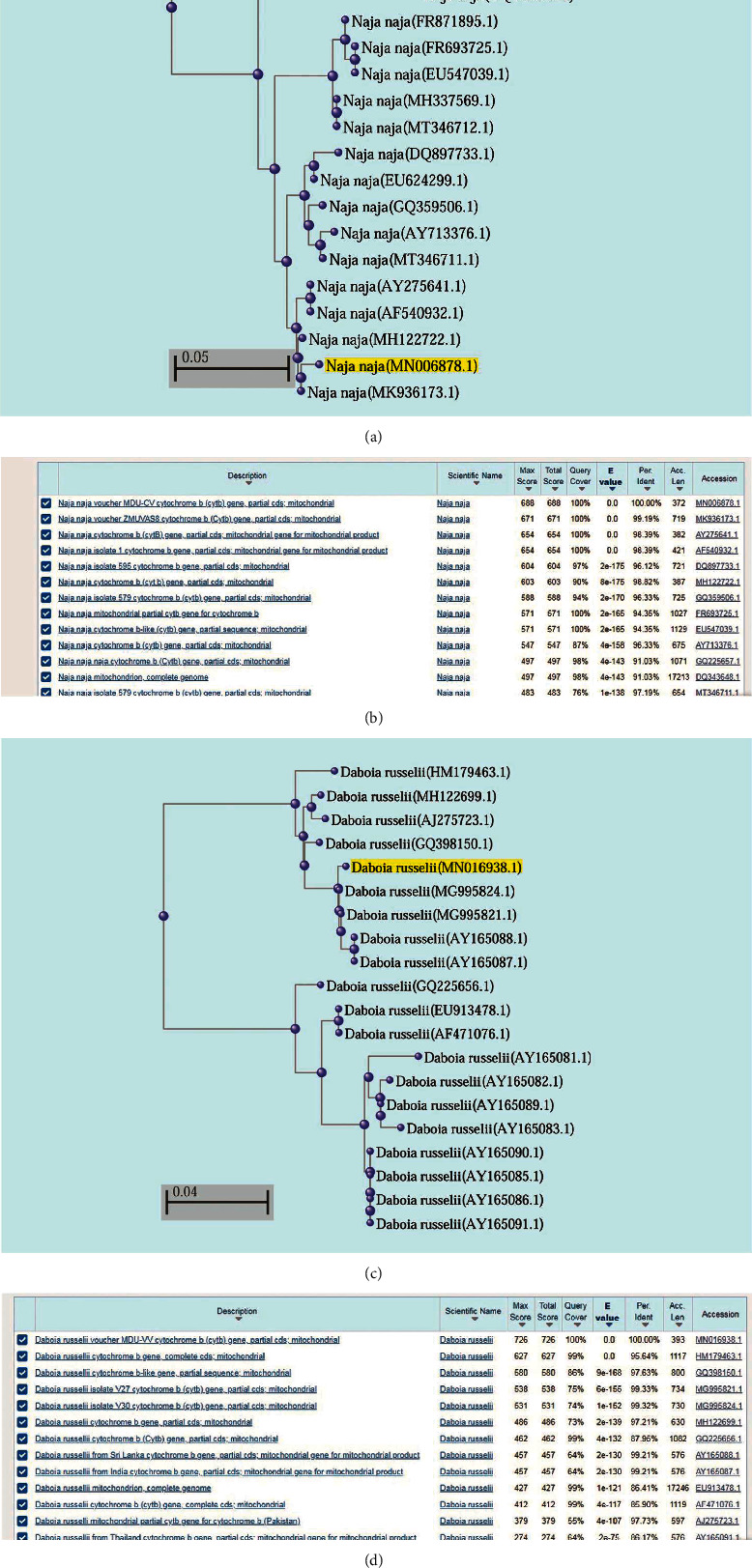
(a, b) Phylogenetic tree of *Naja naja* (a) made using the Neighbour-joining method. Sequence labelling is performed by taxonomic name (Sequence ID) and sequence alignment homology for *Naja naja* query sequence. (b) Sequence highlighted in yellow is query sequence (FASTA sequence is presented in supplementary data file). Phylogenetic tree of *Daboia russelii* (c) made using the Neighbour-joining method. Sequence labelling is performed by taxonomic name (Sequence ID) and sequence alignment homology for *Daboia russelii* query sequence. (d) Sequence highlighted in yellow is query sequence (FASTA sequence is presented in supplementary data file).

Based on nucleotides homology and phylogenetic analysis, the sample cobra (C) was found to be similar to *Naja naja* cytochrome b (Cytb) gene, partial cds; mitochondrial (GenBank Accession Number: MK936173.1) (Figure 3(a)). Other sequences producing significant alignment and homology are shown in Figure 3(b). Based on nucleotide homology and phylogenetic analysis, the sample viper (V) was found to be similar to *Daboia russelii isolate V27 cytochrome b (cytb) gene, partial cds; mitochondrial* (GenBank Accession Number: MG995821.1) (Figure 3(c)). Other sequences producing significant alignment and homology are shown in Figure 3(d).

The accession numbers MN006878 *(Naja naja)* and MN016938 *(Daboia russelii)* were acquired after submitting the query FASTA files to NCBI sequence submitting tool Banklt (https://www.ncbi.nlm.nih.gov/WebSub/) (Supplementary Data File).

### 3.3. SDS-PAGE

**Figure 4:**
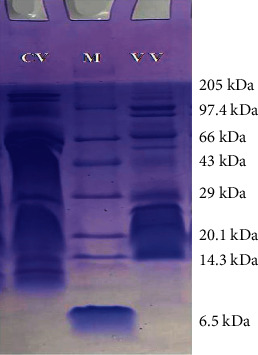
Separation and visualization of proteins bands of venom samples in 12% SDS-PAGE (M, marker protein; CV, cobra venom; VV, viper venom).

The visualization of 12% SDS-PAGE gel showed the separation of proteins over a wide range of molecular weights. The distinguished banding pattern was observed in <10 kDa, 10–30 kDa, 30–50 kDa, >65 kDa regions (Figure 4). Clusters of proteins were observed around 60–66 kDa region in *Naja naja* venom lane and around 26–29 kDa region in *Daboia russelii* venom lane. However, a distinctly different banding pattern in SDS gel is visible, but some bands are broadly shared in venoms.

### 3.4. LC-MS/MS Proteomic Analysis

**Figure 5:**
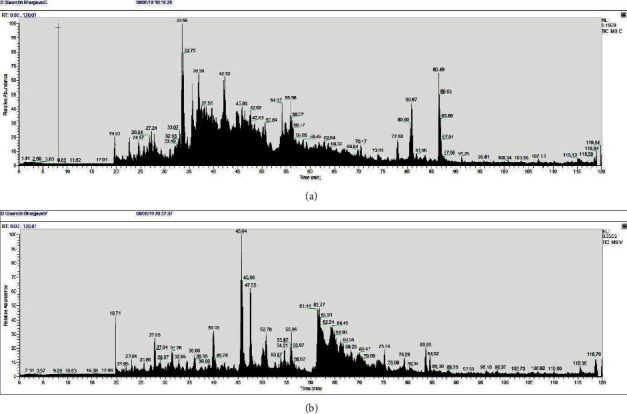
LC-MS/MS chromatograms of *Naja naja* venom (a) and *Daboia russelii* venom (b).

Results of LC-MS/MS analysis in the form of chromatogram are obtained for *Naja naja* venom (Figure (5(a)) and *Daboia russelii* venom (Figure 5(b)). The chromatograms show a clearly distinguished pattern of elution of ions in both samples. The machine parameters were set in a way to predict only high confidence peptides; 188 and 182 peptides of high confidence were detected in *Naja naja* and *Daboia russelii,* respectively. The sequences of these high confidence peptides were searched to find out the homology with proteins of *Serpentes* database of https://www.uniprot.org/

Database searching resulted in finding of proteins and toxins from 12 to 14 protein families in *Naja naja* and *Daboia russelii* venoms. Proteins from seven families were found to be common in venoms of both *Naja naja* and *Daboia russelii*. Five protein/toxin families were exclusively present in *Naja naja* venom, while seven protein/toxin families were present only in *Daboia russelii* venom (Table 2; Supplementary Tables 1–2).

Cytotoxins and neurotoxins of three-finger toxins family in the lower-molecular-weight region (6–9 kDa) were exclusively present in the venom of *Naja naja* only. C-type lectins (16–18 kDa), Snaclecs (18–19 kDa), VEGFs (16–17 kDa), NGFs (27–28 kDa), and disintegrins (4–12 kDa) were exclusively represented in 16–29 kDa region in the venom of *Daboia russelii*. Cobra serum albumin (69.8 kDa) and Cobra venom factor (184.4 kDa) were exclusive to cobra venom, while glutaminyl-peptide cyclotransferases (42.1 kDa) were exclusively present in the venom of *Daboia russelii* only.

### 3.5. ICP-MS Elemental Analysis

Results of elemental analysis using ICP-MS are presented in Table 3. Sodium was present in maximum concentration compared to all elements in both *Naja naja* and *Daboia russelii* venoms, while zinc, magnesium, and calcium were found to be the most abundant divalent cations in both venoms. The difference in relative concentrations of some of the metallic ions was quite prominent in both venoms, while few ions were found to be in comparable limits in both venoms. The concentration of potassium in *Daboia russelii* venom was measured to be around 6.3 times as compared to that in the venom of *Naja naja*. Similarly, the concentration of sodium in viper venom was twice that in cobra venom. The concentrations of zinc, magnesium, and manganese were found to be 7.6, 4.5, and 2 times in cobra venom as compared to that in viper venoms. The concentration of iron ions was marginally more in viper venom, while concentrations of calcium, aluminum, copper, nickel, vanadium, and chromium were almost similar in both venoms.

## 4. Discussion

Seized snake venom is usually found as white yellowish powder form or yellowish viscose liquid form. Powder form can be easily mistaken with any drugs of abuse of similar colour, whereas the liquid form on the other hand, without any physical markers, could be mistaken with plant extracts or other natural/synthetic chemical formulations. It can create technical problems for the law enforcement agencies in framing wildlife charges against the accused [3]. Identification of specific venom antigens in suspected samples and total protein concentration in suspected venom samples is very important for the provisional detection of snake venoms. Positive precipitation reaction with specific antivenom can positively provide direction for further confirmatory testing.

Venom is a cocktail of various protein families. Therefore, protein estimation could be the first step towards screening of suspected snake venom. The protein concentration in venoms of *elapid* and *viperid* snakes is generally expected in the range of 90–92% of dry weight (w/w) of venoms [6]. The results of the present study have also calculated 76.1% and 83.9% protein concentration in the venom of *Naja naja* and *Daboia russelii* snakes, respectively. It is also relatable to mention that venoms samples used in this study were pooled venoms obtained from multiple snakes of the same species. Even the venom extraction/milking method can affect the concentration of proteins in snake venoms [27]. Various other factors including the age, gender, diet, and habitat of snake can also alter the venom composition [28, 29]. Therefore, protein quantification can be used as a preliminary screening for exoneration or inclusion of a suspected material to be snake venom.

The use of the *cyt b* region of mtDNA for species identification is well established in forensic community [24, 30, 31]. Similar kinds of studies have been used to identify snake species also, but in these studies, the source of DNA was meat or scales of the snakes [24]. The use of the mtDNA method from dried snake venom for the identification of snake species was proposed and implemented later [3, 32]. The use of universal primers mcb398 ′TACCATGAGGACAAATATCATTCTG′ and mcb869 “CCTCCTAGTTTGTTAGGGATTGATCG” has proved to be valuable in forensic identifications [3, 24]. The homology of DNA sequences is supposed to be the ultimate evidence in identifying the unknown origin, but there has been a catch point in such studies; the venom being secreted by venomous apparatus, which basically is just a modified parotid gland, is ideally devoid of any source of DNA. The DNA studies are based on assumptions that some buccal cells must have contaminated the saliva while forcefully extracting the venom from live snake. Ideally, venom itself should not contain any cells if ejected by snake itself without any struggle. Therefore, the illegally milked and trafficked venom must have some buccal cells in it, which can be used, as a source of DNA. However, the amount of such contamination is again uncertain. So this method of identifying the source of venom however is accurate but has its own limitation of being based upon the assumptions of contamination while milking, and also the quantity of buccal cells must be enough to be for the isolation of DNA. In addition, this method does not take into consideration about the multiple snakes being milked into the same batch of venom. However, the need of DNA-based evidences in venom identification has earlier been recommended [33].

Higher moleculaer weight based enzymes are more presented in vipers, and lower-molecular-weight nonenzymatic toxins are more common in elapids venom. Disintegrins and myotoxins (4–12 kDa) are visible only in the venom of *Daboia russelii*. CRiSPs (21–29 kDa) and phosphodieterases (>94 kDa) are present in both venoms. In 28–30 kDa region, serine proteases including kallikrein-like, thrombin-like, and arginine esterase are usually present in viperids only and are not presented in elapids. Acetylcholinesterase (55–60 kDa) (only in *Naja naja* venom), prothrombin activators (group-a and d) (45–58 kDa) (only in *Naja naja* venom), snake venom metalloproteinases-p-iii (43–60 kDa), 5′-nucleotidase (53–82 kDa) ,and hyaluronidase (73 kDa) are present in the wide cluster of bands in 45–80 kDa region in SDS gel [28]. The shared protein families among different species indicate evolution from a common ancestry [28]. Observations of the current study are comparable with the earlier studies made on elapids and vipers [34–39]. So, SDS-PAGE can be used to screen an unknown questioned material to know if it is snake venom or not.

Proteomic analysis of study samples carried out with the help of SDS-PAGE is an effective method for comparing these highly cocktailed protein-based venoms. This method is also equally imperative in the coarse identification of snake venom based on identifiable banding patterns. However, SDS alone is not enough to verify a suspected material to be snake venom, but it can corroborate results of other screening tools like the Ouchterlony test or DNA analysis results of *cyt b* region of mtDNA. So, in conjunction with other methods, SDS-PAGE can be used to ascertain the identity of unknown suspected snake venom to help law enforcement agencies framing charges against wildlife crime accused people.

Proteomics of snake venoms has come a long way since the dawn of this century. Advancement in mass spectroscopy has helped to further refine proteomic profiles of snake venoms. Mass spectrometry-based proteomic studies have unravelled the complex proteomes, which lead to better understanding of pathophysiological interpretations of snake envenomation [40–42]. Such proteomic research could also be a potential way in forensic science to identify snake venom up to species level. Earlier studies have proved that elapids venoms are rich in lower-molecular-weight smaller protein and toxins like 3FTXs (three-finger toxins) and type-1 PLA2, while the viper venoms are richly presented by higher-molecular-weight enzymes such as SVMPs, SVSPs, and type-2 PLA2 [6, 28]. The 3FTXs (three-finger toxins) and type-1 PLA2 in snake venoms of elapids could generate a unique pattern for identification. Three-finger toxins (3FTXs) are short- and long-chain nonenzymatic proteins (6-9kDa) abundantly present in cobra venoms, which are mainly neurotoxins and cytotoxins in their roles in elapid snake venoms [17].

PLA2s from group-I subfamily of phospholipase-A2 family exclusively found in the venom of *Naja naja* primarily responsible for localized necrosis and myotoxicity, while from group-II subfamily present only in the venom of *Daboia russelii* are responsible for impairing blood cascade system [43–45]. SVMPs family (20 to 110 kDa) of venom protein contains metalloprotein domain-containing and disintegrin domain-containing proteins. These are abundantly present in viper venoms and are in lesser concentrations in cobra venoms [6, 17]. The main effects of these metal-dependent enzymes are hemorrhage and coagulopathy [6, 17, 46]. SVSPs of serine protease family (26–67 kDa) are more abundant in viper venoms as compared to cobra venoms [17]. In the present study, LC-MS/MS profiles of venoms were analogous to earlier studies [28, 34, 37, 43, 44, 47, 48]. It is now well understood that such distinct proteomes of both species can be used to identify and authenticate the venoms milked from them and can verify the species of origin of venoms.

However, the nature of snake venoms is highly multifaceted, but one thing is well established that the major actions of venoms on its prey are due to organic components only. The inorganic constituents of snake venoms including the metallic ions are sparsely studied that too, not in recent years. Studies regarding the role of metals in the mechanism of action of snake venoms were attempted in the 1960s and 1970s. Neutron activation analysis (NAA) of snake venoms showed the presence of copper, zinc, magnesium, sodium, and potassium in varying amount in different species [49, 50]. Elemental analysis using atomic absorption spectrometry (AAS) of venoms was performed for sodium, potassium, calcium, magnesium, copper, manganese, iron, cobalt, zinc, bismuth, selenium, platinum, palladium, molybdenum, arsenic, and gold ions [51, 52].

Role of metals in snake venoms is yet to be ascertained completely, but there have been reports that suggest that some metallic ions have a say in toxicity and hemorrhage activity of venoms. The addition of chelating agents have resulted reduced toxicity of venoms [53–55]. Removal of calcium has resulted in decreased hemorrhagic activity, thereby decreasing the lethality of venom [56, 57]. Similar results were obtained for magnesium ions also. The role of metallic ions has been suggested to alter the hemorrhagic and proteolytic activities of venoms. Magnesium has been proved to assist in causing hemorrhagic activities. Copper and zinc were found in higher concentrations in neurotoxic and hemorrhagic fractions [58]. The exact role of metals in the propagation of venom-induced effects is yet to be studied extensively. However, the correlation between diet of a snake and its toxicity has not been established, but on the contrary, there were found many changes in the toxicity of snakes of different prey eaters [28]. The venoms for this study were obtained from the same place, where snakes are kept in a similar environment and are fed a similar kind of feed nullifying any difference in diet, and yet, there are significant differences in concentrations of many metallic ions, and this proves that there might be some role of these ions in the mechanism of action of venoms in tissues of its prey. Extensive research on the elemental analysis of venoms of multiple snakes of different species from different regions is required for in-depth knowledge of their roles in venom actions.

Higher concentrations of metals can be related to the presence of such enzymes, which need those particular metallic ions as cofactors in their catalytic activities. PLA2s are abundantly presented in both venoms, and their enzymatic function is carried out with calcium as cofactor. The quantity of calcium is found to be almost similar in both venoms. Cobra venom factor (CVF) activates complement resulting in the depletion of complement activity. This activity of CVF is carried out in the presence of magnesium ions (Mg^++^). Magnesium is found in a higher (4.5X) quantity in cobra venom than viper venom. The quantity of zinc is found quite high (7.5X) in cobra venom as compared to viper venom. Zinc ions (Zn^++^) act as cofactors in number of venom enzymes including SVMPs, nucleotidases, and cobra venom factor. SVMPs and nucleotidases are detected in both venoms, but the cobra venom factor is exclusive to cobra venom. Phosphodiesterase (PDE) uses divalent metallic ions as cofactors in disseminating their enzymatic functions. In our findings, only cobra venom was found to contain PDE. Cobra venom contains higher quantities of almost all studied metals that usually exist in divalent cation form and acts as cofactors to catalytic activities of venom enzymes (https://www.uniprot.org/uniprot/C0HK16).

Such studies were never carried out in forensic prospective, but the current research attempt was a significant step in order to quantitatively analyze trace elemental profiles (if any) on venoms of *Naja naja* and *Daboia russelii* for the purpose of identification and comparison of such samples. A significant difference in concentrations of many metallic ions could prove some role of these ions in the mechanism of action of venoms in tissues of its prey. Extensive research on the elemental analysis of venoms of multiple snakes of different species from different regions is required for in-depth knowledge of their roles in venom actions.

## 5. Conclusion

**Figure 6:**
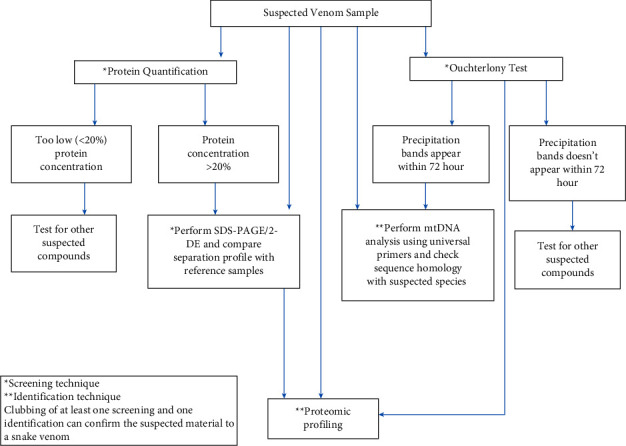
The proposed template for screening and identification of suspected snake venoms in forensic labs.

Although most of the techniques described in this article are generally practiced in snake venom research, their forensic use in solving wildlife-related crimes of illegal snake venom trafficking is atypical and not standardized. The law enforcement agencies in wildlife-related crime have to remain dependent on scientific expertise in proving the identification and verification of seized snake venom samples. The lack of uniformity in dealing with snake venom analysis in forensic science laboratories results in a higher acquittal rate, thereby indirectly encouraging criminals in cash-rich illegal trafficking of snake venoms. The need of the hour is to standardize the protocol for snake venom analysis in forensic labs. This research attempted to describe screening as well as confirmatory tools for analyzing suspected snake venoms. Furthermore, advanced instrumental methods such as LC-MS/MS and ICP-MS could also be of immense help in such investigations for the experts. Clubbing of two or more scientific techniques can be performed by forensic experts to corroborate the results in proving the identification of snake venoms. The proposed template is shown in Figure 6. This study, however, should not be considered final compendium because it is a preliminary attempt involving venom samples of only two snake species. It is conceivable that there may still be situations in which this scientific report could be of potential use.

### 5.1. Limitation of Study

This study of venom identification and characterization was conducted on venoms procured from an authorized seller. However, in real-life cases, forensic samples of suspected venoms could be lesser in amount, more degraded or mixtures of multiple snake species. Therefore, the use of these techniques on real-world forensic samples will show the actual benefit of clubbing of these techniques. Being in an academic institute, we could not get the seized samples for analysis, but we expect researchers in forensic science labs to try this template and verify the success of these techniques in wildlife crimes involving snake venom analysis.

## Figures and Tables

**Table 1:** Details of various machine and experiment parameters of LC-MS/MS analysis of snake venoms.

General reverse-phase nano-liquid chromatography parameters	General Mass spectrometer parameters	Database searching parameters
Instrument name: Thermo Scientific Easy-nLC 1200	Instrument name: Thermo Scientific Q Exactive Orbitrap	Software used: Proteome Discoverer 2.2
(i) Amount of peptide loaded on column: ∼3 *µ*g (ii) Analytical column name: PepMap RSLC C18 2*µ*m × 50 cm (Thermo scientific) (iii) Column temperature: 40^0^ C	*General setting:* (i) Run time: 0 to 140 min (ii) Polarity: Positive (iii) Default charge state: 2 *MS (MS1) setting:* (i) Microscan: 1 (ii) Resolution: 70000 (iii) AGC (Automatic gain control) 3e^6^ (iv) Maximum IT (ion transfer) time: 50 ms (v) No. of scan ranges: 1 (vi) Scan ranges: 350 to 1700m/z (vii) Spectrum obtained was in profile mode *MS MS (MS2) setting:* (i) Microscan: 1 (ii)Resolution: 17500 (iii)AGC (Automatic gain control) 2e^5^ (iv)Maximum IT (ion transfer) 100 ms (v) Loop count: 15 (top 15 masses will be fragmented one by one) (vi) Maximum number of precursor to be plexed in single event: 1 (vii) Isolation window: 1.2 m/z (viii) Isolation offset: 0.0 m/z (ix) Fixed first mass: 100 m/z (x) Normalized collision energy: 27 (xi) *Data dependent settings* (xii) Minimum AGC target: 1.20e^2^ (xiii) Charge exclusion: unassigned, 1, 7, 8, >8 (xiv) Dynamic exclusion time: 50.0 S	Database of *SERPENTES* was directly downloaded from http://www.uniprot.org in fasta format. (i) Maximum allowed missed cleavage: 2 (ii) Minimum peptide length for search: 2 (iii) Maximum peptide length: 144 (iv) Precursor mass tolerance: 10 ppm (v) Fragment mass tolerance: 0.02 Da (vi) Dynamic modification: Oxidation of Methionine and Acetylation at N terminus (vii) Static modification: Carbamidomethylation (viii) Target FDR (false discovery rate): 0.01 (for decoy database search) (ix) Validation was based on q value.

**Table 2:** Various types of proteins found in LC-MS/MS of crude venoms of *Naja naja* and *Daboia russelii.*

Sr. No.	Protein/toxin	*Naja naja* venom	*Daboia russelii* venom
1.	Phospholipases (PLA2)	Yes	Yes
2.	Snake venom metalloproteases (SVMPs)	Yes	Yes
3.	Snake venom serine proteinases (SVSPs)	Yes	Yes
4.	Cysteine-rich secretory proteins (CRiSPs)	Yes	Yes
5.	Kunitz-type serine protease inhibitor	Yes	Yes
6.	L-Amino acid oxidases (LAAOs)	Yes	Yes
7.	Nucleotidase	Yes	Yes
8.	Three-finger toxins (3FTXs)	Yes	No
9.	Snake venom phosphodiesterase (PDE)	Yes	No
10.	Acetylcholinesterase	Yes	No
11.	Cobra venom factor	Yes	No
12.	Cobra serum albumin	Yes	No
13.	Snaclec/C-type lectin	No	Yes
14.	Disintegrins (Dis)	No	Yes
15.	Nerve growth factor (NGF)	No	Yes
16.	Vascular endothelial growth factor (VEGF)	No	Yes
17.	Glutaminyl-peptide cyclotransferases	No	Yes
18.	78 kDa glucose-regulated protein	No	Yes
19.	Keratin, type II cytoskeletal	No	Yes

**Table 3:** Quantitative elemental analysis of *Naja naja* and *Daboia russelii* venoms using ICP-MS. Na, sodium; K, potassium; Ca, calcium; Mg, magnesium; Al, aluminum; V, vanadium; Cr, chromium; Mn, manganese; Fe, iron; Co, cobalt; Ni, nickel; Cu, copper; Zn, zinc; ppm , parts per million; RSD, relative standard deviation.

Name of element	*Naja naja* venom (ppm)	RSD (%)	*Daboia russelii* venom (ppm)	RSD (%)
Na	9192.8689	0.9	18887.6104	1.4
K	955.6169	1.8	6108.1359	3.3
Ca	362.9142	0.7	361.4635	3.9
Mg	2745.2567	0.7	601.0782	1.6
Al	116.6738	2.2	122.8210	2
V	0.3049	5.5	0.3059	0.6
Cr	2.7004	1.7	2.8973	1.5
Mn	9.3255	0.8	4.2961	2.2
Fe	128.7793	0.3	166.0583	3.6
Co	0.1222	1.9	0.0951	3
Ni	1.7695	0.3	2.121	2.1
Cu	3.4218	1.4	3.6992	0.9
Zn	5317.9606	1.2	699.3199	1.6

## Data Availability

Proteomic data (LC-MS/MS data) from this work can be accessed at https://data.mendeley.com/datasets/g6xz3j6hp8/1 and is also available in supplementary files (Figures 1 and 2(a)–2(d) and Tables 2–4). Sequence homology of DNA regions submitted in GenBank can be accessed at https://www.ncbi.nlm.nih.gov (https://www.ncbi.nlm.nih.gov/nuccore/MN006878.1?report=GenBank; https://www.ncbi.nlm.nih.gov/nuccore/MN016938.1?report=GenBank).

## Supplementary Materials

Supplementary Materials Supplementary figure 1: (a) electropherogram with DNA bands; (b) agarose gel of PCR product of sample; (c) cobra (Naja naja) venom; V :  viper (*Daboia russelii*) venom; L :  DNA ladder. Supplementary table 1: summary of various proteins homology identified with peptide sequences generated from LC-MS/MS analysis of *Naja naja*. Supplementary table 2: summary of various proteins homology identified with peptide sequences generated from LC-MS/MS analysis of *Daboia russelii*.
