# Supplementary material for: Comparative Snake Venom Analysis for Facilitating Wildlife Forensics: A Pilot Study
Source: J Anal Methods Chem. 2022 Jun 3;2022:8644993. doi: 10.1155/2022/8644993 (PMC9187493; doi:10.1155/2022/8644993)
Supplement: Supplementary Materials — Supplementary figure 1: (a) electropherogram with DNA bands; (b) agarose gel of PCR product of sample; (c) cobra (Naja naja) venom; V : viper (Daboia russelii) venom; L : DNA ladder. Supplementary table 1: summary of various proteins homology identified with peptide sequences generated from LC-MS/MS analysis of Naja naja. Supplementary table 2: summary of various proteins homology identified with peptide sequences generated from LC-MS/MS analysis of Daboia russelii. [file 8644993.f1.docx]

**Supplementary data**

Detailed results of LC-MS/MS analysis of *Naja naja* **(Supplementary Table-1)** venom.

| **Supplementary Table 1:** Summary of various proteins homology identified with peptide sequences generated from LC-MS/MS analysis of *Naja naja*. | | | | | | | | |
| --- | --- | --- | --- | --- | --- | --- | --- | --- |
| **Protein Family** | **Protein Description** | **Accession** | **Coverage [%]** | **Homology with protein from** | **# PSMs** | **# Unique Peptides** | **MW [kDa]** | **Score SequestHT: SequestHT** |
| **Three Finger Toxins (3FTXs)**  **(16)** | Cytotoxin homolog 5V | Q9W716 | 25 | *Naja atra* | 7 | 3 | 9.3 | 15.71 |
|  | Cardiotoxin 7'' | Q91996 | 25 | *Naja atra* | 7 | 3 | 9.3 | 15.71 |
|  | Cytotoxin-like basic protein | P62377 | 34 | *Naja naja* | 7 | 3 | 7 | 15.71 |
|  | Cardiotoxin7a | Q91126 | 25 | *Naja atra* | 7 | 3 | 9.3 | 15.71 |
|  | CytotoxinA5 | P62375 | 25 | *Naja atra* | 7 | 3 | 9.3 | 15.71 |
|  | Cytotoxin homolog 5 | Q91137 | 25 | *Naja atra* | 7 | 3 | 9.3 | 15.71 |
|  | Long neurotoxin 1 | P25668 | 32 | *Naja naja* | 2 | 2 | 7.8 | 4.31 |
|  | Long neurotoxin 2 | P25669 | 32 | *Naja naja* | 2 | 2 | 7.8 | 4.31 |
|  | Cytotoxin 7 | P49122 | 11 | *Naja atra* | 4 | 1 | 9.1 | 8.34 |
|  | Cytotoxin 11 | P62394 | 18 | *Naja haje haje* | 2 | 1 | 6.8 | 4.4 |
|  | Cytotoxin homolog | P14541 | 18 | *Naja kaouthia* | 2 | 1 | 7 | 4.4 |
|  | Long neurotoxin 5 | P25673 | 14 | *Naja naja* | 1 | 1 | 7.9 | 2.47 |
|  | Long neurotoxin 4 | P25672 | 14 | *Naja naja* | 1 | 1 | 7.9 | 2.47 |
|  | Long neurotoxin 3 | P25671 | 14 | *Naja naja* | 1 | 1 | 7.8 | 2.47 |
|  | Muscarinic toxin-like protein 3 | P82464 | 15 | *Naja kaouthia* | 1 | 1 | 7.6 | 2.67 |
|  | Neurotoxin homolog NL1 | Q9DEQ3 | 12 | *Naja atra* | 1 | 1 | 10 | 2.67 |
|  | | | | | | | | |
| **Phospholipases (PLA2s)**  **(10)** | Acidic phospholipase A2 2 | P15445 | 47 | *Naja naja* | 12 | 2 | 13.3 | 23.98 |
|  | Acidic phospholipase A2 E | P25498 | 29 | *Naja oxiana* | 11 | 2 | 13.2 | 23.98 |
|  | Neutral phospholipase A2 muscarinic inhibitor | Q92084 | 32 | *Naja sputatrix* | 10 | 1 | 16.2 | 22.22 |
|  | Acidic phospholipase A2 C | Q92086 | 32 | *Naja sputatrix* | 10 | 1 | 16.1 | 22.22 |
|  | Acidic phospholipase A2 D | Q9I900 | 32 | *Naja sputatrix* | 10 | 1 | 16.1 | 22.22 |
|  | Neutral phospholipase A2 B | Q92085 | 32 | *Naja sputatrix* | 10 | 1 | 16.2 | 22.22 |
|  | Acidic phospholipase A2 4 (Fragment) | Q6T179 | 34 | *Naja sagittifera* | 10 | 1 | 14.2 | 22.24 |
|  | Acidic phospholipase A2 1 | P00596 | 18 | *Naja kaouthia* | 9 | 1 | 16.3 | 22.22 |
|  | Acidic phospholipase A2 3 (Fragment) | P60045 | 17 | *Naja sagittifera* | 9 | 1 | 14 | 22.24 |
|  | Acidic phospholipase A2 5 (Fragment) | Q5G291 | 25 | *Naja sagittifera* | 2 | 1 | 13.8 | 2.24 |
|  | | | | | | | | |
| **Snake Venom Metalloproteinases**  **(SVMPs)**  **(6)** | Zinc metalloproteinase-disintegrin-like cobrin | Q9PVK7 | 17 | *Naja kaouthia* | 20 | 4 | 67.6 | 41.94 |
|  | Zinc metalloproteinase-disintegrin-like atrase-B | D6PXE8 | 12 | *Naja atra* | 39 | 3 | 66.2 | 55.98 |
|  | Zinc metalloproteinase-disintegrin-like kaouthiagin-like | D3TTC1 | 12 | *Naja atra* | 39 | 3 | 66.2 | 55.98 |
|  | Zinc metalloproteinase-disintegrin-like atragin | D3TTC2 | 11 | *Naja atra* | 11 | 1 | 69.1 | 26.08 |
|  | Hemorrhagic metalloproteinase-disintegrin-like kaouthiagin | P82942 | 18 | *Naja kaouthia* | 38 | 3 | 44.5 | 55.29 |
|  | Zinc metalloproteinase-disintegrin-like atrase-A | D5LMJ3 | 7 | *Naja atra* | 5 | 4 | 68.2 | 14.61 |
|  | | | | | | | | |
| **Snake Venom Serine Proteases (SVSPs)**  **(4)** | Serine proteinase 1a | A0A194AT39 | 7 | *Micrurus tener* | 1 | 1 | 29.1 | 3.37 |
|  | Serine protease harobin | Q5MCS0 | 7 | *Hydrophish ardwickii* | 1 | 1 | 29 | 3.37 |
|  | Snake venom serine protease NaSP (Fragment) | A8QL53 | 7 | *Naja atra* | 1 | 1 | 31.1 | 3.37 |
|  | Serine proteinase 1c | A0A194AP49 | 7 | *Micrurus tener* | 1 | 1 | 29.2 | 3.37 |
|  | | | | | | | | |
| **Cysteine Rich Secretory Proteins (CRiSPs)**  **(7)** | Cysteine-rich venom protein (Fragment) | P86543 | 70 | *Naja naja* | 14 | 2 | 3.9 | 32.1 |
|  | Cysteine-rich venom protein annuliferin-b (Fragment) | P0DL15 | 77 | *Naja annulifera* | 14 | 2 | 3.6 | 32.1 |
|  | Cysteine-rich venom protein natrin-1 | Q7T1K6 | 15 | *Naja atra* | 5 | 2 | 26.9 | 10.86 |
|  | Cysteine-rich venom protein kaouthin-1 | P84805 | 15 | *Naja kaouthia* | 5 | 2 | 26.8 | 10.86 |
|  | Cysteine-rich venom protein annuliferin-a (Fragment) | P0DL14 | 37 | *Naja annulifera* | 11 | 2 | 3.6 | 23.19 |
|  | Cysteine-rich venom protein mossambin (Fragment) | P0DL16 | 37 | *Naja mossambica* | 11 | 2 | 3.6 | 23.19 |
|  | Cysteine-rich venom protein 25 (Fragment) | P84806 | 44 | *Naja haje haje* | 11 | 2 | 3 | 23.19 |
|  | | | | | | | | |
| **L-amine Acid Oxidase (LAAO)**  **(8)** | Amine oxidase (Fragment) | A0A2R4N4Q6 | 30 | *Naja atra* | 46 | 19 | 57.9 | 85.65 |
|  | L-amino-acid oxidase (Fragment) | A8QL58 | 26 | *Naja atra* | 38 | 15 | 51.4 | 70.15 |
|  | L-amino-acid oxidase OS= scutellatus | Q4JHE3 | 10 | *Oxyuranus scutellatus* | 20 | 8 | 59 | 39.46 |
|  | Amine oxidase (Fragment) | R4FJP5 | 8 | *Echiopsis curta* | 17 | 7 | 59.2 | 31.6 |
|  | Amino_oxidase domain-containing protein (Fragment) | A0A2D4Q0B3 | 32 | *Micrurus surinamensis* | 13 | 6 | 11.1 | 23.18 |
|  | L-amino-acid oxidase (Fragments) | P0DI91 | 34 | *Naja oxiana* | 5 | 4 | 11.2 | 11.33 |
|  | L-amino-acid oxidase | A8QL51 | 6 | *Bungarus multicinctus* | 7 | 3 | 58.8 | 17.38 |
|  | Amine oxidase | A0A194ARE6 | 6 | *Micrurus tener* | 7 | 3 | 58.7 | 17.38 |
|  | | | | | | | | |
| **Kunitz Type Serine Protease Inhibitor**  **(1)** | Kunitz-type serine protease inhibitor | P20229 | 39 | *Naja naja* | 9 | 2 | 6.4 | 25.09 |
|  | | | | | | | | |
| **Nucleotidase**  **(6)** | Ecto-5'-nucleotidase | A0A194AS98 | 16 | *Micrurus tener* | 7 | 7 | 63 | 15.19 |
|  | Ecto-5'-nucleotidase 1c | U3FYP9 | 16 | *Micrurus fulvius* | 7 | 7 | 62.9 | 15.19 |
|  | Ecto-5'-nucleotidase 1a | A0A0F7YZM6 | 16 | *Micrurus fulvius* | 7 | 7 | 63 | 15.19 |
|  | Venom 5'-nucleotidase | A0A2I4HXH5 | 12 | *Naja atra* | 6 | 6 | 58.2 | 12.52 |
|  | 5'-nucleotidase (Fragment) | W8EFS0 | 14 | *Macro vipera lebetina* | 5 | 5 | 45 | 12.52 |
|  | 5nucleotidase (Fragment) | A0A182C5T8 | 11 | *Phalotris mertensi* | 5 | 5 | 57.5 | 12.52 |
|  | | | | | | | | |
| **Miscellaneous**  **(4)** | Snake venom phosphodiesterase (PDE) | A0A2D0TC04 | 7 | *Naja atra* | 6 | 6 | 94.6 | 9.2 |
|  | Acetylcholinesterase | Q92035 | 5 | *Bungarus fasciatus* | 3 | 1 | 68 | 8.52 |
|  | Cobra serum albumin | Q91134 | 9 | *Naja naja* | 4 | 4 | 69.8 | 7.59 |
|  | Cobra venom factor | Q91132 | 31 | *Naja kaouthia* | 99 | 32 | 184.4 | 159.27 |

Detailed results of LC-MS/MS analysis of *Daboia russelii* **(Supplementary Table-2)** venom.

| **Supplementary Table 2:** Summary of various proteins homology identified with peptide sequences generated from LC-MS/MS analysis of *Daboia russelii*. | | | | | | | | |
| --- | --- | --- | --- | --- | --- | --- | --- | --- |
| **Protein Family** | **Protein Description** | **Accession** | **Coverage [%]** | **Homology with protein from** | **# PSMs** | **# Unique Peptides** | **MW [kDa]** | **Score SequestHT: SequestHT** |
| **Phospholipases (PLA2)**  **(7)** | Basic phospholipase A2daboxin P | C0HK16 | 19 | *Daboia russelii* | 21 | 4 | **13.6** | 40.48 |
|  | Basic phospholipase A2VRV-PL-VIIIa | P59071 | 19 | *Daboia russelii* | 21 | 4 | **13.6** | 40.48 |
|  | Basic phospholipase A2 3 | P86368 | 12 | *Daboia russelii* | 18 | 1 | **13.7** | 8.5 |
|  | Basic phospholipase A2VRV-PL-V | P84674 | 12 | *Daboia russelii* | 18 | 1 | **13.6** | 8.5 |
|  | Basic phospholipase A2DsM-S1 | A8CG84 | 12 | *Daboia siamensis* | 12 | 2 | **15.4** | 25.39 |
|  | Phospholipase A2 | B3RFI8 | 12 | *Daboia russelii limitis* | 12 | 2 | **15.3** | 25.39 |
|  | Phospholipase A2bitanarin (Fragments) | P0DKT6 | 20 | *Bitis arietans* | 7 | 1 | **3.8** | 15.08 |
|  | | | | | | | | |
| **Snake Venom Metalloproteases**  **(SVMPs)**  **(7)** | RVV-X heavy chain OS=Daboiasiamensis OX=343250 PE=2 SV=1 | A0A2H4Z2W1 | 6 | *Daboia siamensis* | 3 | 1 | **69.3** | 7.48 |
|  | SVMP-Cau1 (Fragment) | A7X4X9 | 18 | *Causus rhombeatus* | 1 | 1 | **10.3** | 2.99 |
|  | DSAIP (Fragment) | A0A2H4Z2X4 | 6 | *Daboia siamensis* | 3 | 1 | **69.1** | 6.79 |
|  | DSAIP (Fragment) | A0A2H4Z2Y9 | 6 | *Daboia siamensis* | 2 | 1 | **54.4** | 5.08 |
|  | Metalloproteinase type III 7a | A0A194AS35 | 3 | *Sistrurus miliarius barbouri* | 1 | 1 | **68.4** | 2.28 |
|  | Metalloproteinase type III 7b | A0A194ATS1 | 3 | *Sistrurus miliarius barbouri* | 1 | 1 | **68.3** | 2.28 |
|  | Factor X activator heavy chain | K9JAW0 | 3 | *Daboia russelii russelii* | 2 | 1 | **69.5** | 4.37 |
|  | | | | | | | | |
| **Snake Venom Serine Proteinases (SVSPs)**  **(11)** | Factor V activator RVV-V alpha | P18964 | 50 | *Daboia siamensis* | 39 | 12 | **26.2** | 98.92 |
|  | Factor V activator RVV-V gamma | P18965 | 39 | *Daboia siamensis* | 37 | 11 | **28.8** | 98.92 |
|  | Alpha-fibrinogenase-like | E5L0E3 | 24 | *Daboia siamensis* | 6 | 4 | **28.5** | 20.29 |
|  | Factor V activator | Q9PT41 | 5 | *Macrovipera lebetina* | 19 | 3 | **28.6** | 47.38 |
|  | Beta-fibrinogenase-like | E5L0E4 | 14 | *Daboia siamensis* | 11 | 2 | **28** | 25.79 |
|  | Serine proteinase 10 | A0A0K8RYQ4 | 5 | *Crotalus horridus* | 4 | 2 | **28.8** | 10.6 |
|  | Venom serine proteinase-like protein 2 | Q9PT40 | 7 | *Macrovipera lebetina* | 3 | 1 | **28.9** | 5.77 |
|  | Vaa serine proteinase homolog 1 | A0A1I9KNP0 | 7 | *Vipera ammodytesammodytes* | 3 | 1 | **28.9** | 5.77 |
|  | Serine protease 4 | A0A193CHJ0 | 4 | *Crotalus tzabcan* | 2 | 1 | **29.1** | 5.59 |
|  | Snake venom serine protease | Q2QA04 | 4 | *Crotalus durissusdurissus* | 2 | 1 | **28.3** | 5.59 |
|  | Serine protease VLSP-3 | E0Y420 | 12 | *Macrovipera lebetina* | 2 | 1 | **28.3** | 5.34 |
|  | | | | | | | | |
| **Snaclec/C-type lectin**  **(22)** | Dabocetin beta subunit | K9JDJ6 | 25 | *Daboia siamensis* | 10 | 4 | **18.1** | 19.52 |
|  | Dabocetin beta subunit | K9JDE8 | 25 | *Daboia russelii* | 10 | 4 | **18** | 19.52 |
|  | p31 beta subunit | K9JDF6 | 28 | *Daboia siamensis* | 3 | 2 | **17.3** | 6.15 |
|  | p31 beta subunit | K9JDK6 | 28 | *Daboia russelii* *russelii* | 3 | 2 | **17.4** | 6.15 |
|  | C-type lectin-like protein 4B | A0A0C5DGP5 | 12 | *Macrovipera lebetina* | 2 | 1 | **17.1** | 6.15 |
|  | p31 beta subunit | K9JBV3 | 12 | *Daboia* *russelii* *limitis* | 2 | 1 | **17.2** | 6.15 |
|  | Snaclecdabocetin subunit alpha | Q38L02 | 25 | *Daboia siamensis* | 3 | 3 | **17.5** | 5.51 |
|  | Dabocetin alpha subunit | K9JBU0 | 25 | *Daboia* *russelii* *russelii* | 3 | 3 | **17.5** | 5.51 |
|  | p68 alpha subunit | K9JDF2 | 13 | *Daboia russelii* *limitis* | 2 | 2 | **18.1** | 2.83 |
|  | p68 alpha subunit | K9JBV0 | 13 | *Daboia siamensis* | 2 | 2 | **18** | 2.83 |
|  | C-type lectin snaclec-1 | A0A1I9KNP6 | 6 | *Vipera ammodytesammodytes* | 2 | 1 | **17.6** | 5.14 |
|  | Snaclec A13 | B4XSY8 | 8 | *Macrovipera lebetina* | 2 | 1 | **15.3** | 5.14 |
|  | Snaclec A12 | B4XSY7 | 6 | *Macrovipera lebetina* | 2 | 1 | **17.7** | 5.14 |
|  | Snacleclebecin subunit alpha | W5XDM0 | 7 | *Macrovipera lebetina* | 2 | 1 | **17.2** | 5.14 |
|  | Snaclec A16 | B4XSZ1 | 6 | *Macroviper alebetina* | 2 | 1 | **17.8** | 5.14 |
|  | Snaclec 5 | Q4PRC8 | 13 | *Daboia siamensis* | 3 | 2 | **17.1** | 2.91 |
|  | Snaclec 3 | Q4PRD0 | 13 | *Daboia siamensis* | 3 | 2 | **16.9** | 2.91 |
|  | C-type lectin snaclec-3 | A0A1I9KNN1 | 6 | *Vipera ammodytesammodytes* | 1 | 1 | **17.4** | 3.27 |
|  | C-type lectin-like protein 2A | A0A0C5DKK6 | 6 | *Macrovipera lebetina* | 1 | 1 | **17.7** | 3.27 |
|  | C-type lectin-like protein 2B | A0A0C5DQX8 | 6 | *Macrovipera lebetina* | 1 | 1 | **17.4** | 2.29 |
|  | p31 alpha subunit | K9JDK1 | 6 | *Daboia russelii* *limitis* | 2 | 1 | **18.2** | 2.12 |
|  | p31 alpha subunit | K9JCR7 | 6 | *Daboia russelii* *russelii* | 2 | 1 | **18.1** | 2.12 |
|  | | | | | | | | |
| **Cysteine Rich Secretory Proteins (CRiSPs)**  **(10)** | Cysteine-rich seceretory protein Ch-CRPKc (Fragment) | F2Q6E7 | 18 | *Crotalus horridus* | 6 | 3 | **24.8** | 11.76 |
|  | Cysteine-rich seceretory protein Ch-CRPIa (Fragment) | F2Q6E8 | 18 | *Crotalus horridus* | 6 | 3 | **24.7** | 11.76 |
|  | Cysteine-rich seceretory protein Ch-CRPIb (Fragment) | F2Q6E9 | 18 | *Crotalus horridus* | 6 | 3 | **24.7** | 11.76 |
|  | Cysteine-rich seceretory protein Ch-CRPKb (Fragment) | F2Q6E6 | 18 | *Crotalus horridus* | 6 | 3 | **24.7** | 11.76 |
|  | Cysteine-rich seceretory protein Ch-CRPKa (Fragment) | F2Q6E5 | 18 | *Crotalus horridus* | 6 | 3 | **24.7** | 11.76 |
|  | Cysteine-rich seceretory protein Dr-CRPK | F2Q6F2 | 12 | *Daboia russelii* | 3 | 1 | **26.7** | 10.41 |
|  | Cysteine-rich seceretory protein Dr-CRPB (Fragment) | F2Q6F3 | 10 | *Daboia russelii* | 2 | 1 | **25** | 5.16 |
|  | Cysteine-rich seceretory protein Cv-CRP (Fragment) | F2Q6F1 | 10 | *Crotalus viridis* | 2 | 1 | **24.8** | 5.16 |
|  | Cysteine-rich venom protein Bco13 (Fragment) | P0DMG5 | 67 | *Bothrops cotiara* | 1 | 1 | **1.7** | 2.41 |
|  | Cysteine-rich venom protein (Fragment) | P86537 | 100 | *Daboia russelii* | 1 | 1 | **1.1** | 2.41 |
|  | | | | | | | | |
| **Kunitz type serine protease inhibitor**  **(7)** | Kunitz-type serine protease inhibitor C4 | A8Y7N7 | 11 | *Daboia siamensis* | 3 | 2 | **10.2** | 6.13 |
|  | Kunitz-type protease inhibitor | H9BFA3 | 11 | *Daboia russelii* | 3 | 2 | **10.1** | 6.13 |
|  | Kunitz-type serine protease inhibitor B5 | A8Y7P5 | 11 | *Daboia siamensis* | 3 | 2 | **9.9** | 6.13 |
|  | Kunitz-type serine protease inhibitor C3 | A8Y7N6 | 12 | *Daboia siamensis* | 3 | 2 | **9.4** | 6.13 |
|  | Kunitz-type serine protease inhibitor B1 | A8Y7P1 | 12 | *Daboia siamensis* | 3 | 2 | **9.3** | 6.13 |
|  | Kunitz-type serine protease inhibitor DrKIn-I | H6VC05 | 11 | *Daboia russelii* | 1 | 1 | **10** | 2.49 |
|  | Kunitz-type serine protease inhibitor B6 | A8Y7P6 | 12 | *Daboia siamensis* | 1 | 1 | **9.3** | 2.49 |
|  | | | | | | | | |
| **L-amino Acid Oxidases (LAAOs)**  **(7)** | L-amino-acid oxidase | G8XQX1 | 51 | *Daboia russelii* | 117 | 15 | **56.9** | 228.67 |
|  | L-amino-acid oxidase (Fragments) | Q4F867 | 45 | *Daboia siamensis* | 75 | 11 | **46.3** | 132.54 |
|  | L-amino-acid oxidase (Fragments) | P0C2D7 | 50 | *Vipera berus berus* | 35 | 1 | **10.3** | 38.18 |
|  | L-amino-acid oxidase | P0DI84 | 10 | *Vipera ammodytesammodytes* | 6 | 1 | **54.7** | 15.4 |
|  | L-amino acid oxidase (Fragment) | X2L4E2 | 6 | *Bothrops pictus* | 5 | 1 | **56.3** | 12.37 |
|  | L-amino acid oxidase 1d | A0A194ATK4 | 4 | *Sistrurus tergeminus* | 2 | 1 | **58.7** | 3.79 |
|  | L-amino acid oxidase 1a | A0A194APS7 | 4 | *Sistrurus tergeminus* | 2 | 1 | **58.8** | 3.79 |
|  | | | | | | | | |
| **Disintegrins (Dis)**  **(2)** | Disintegrin CV | Q3BK17 | 23 | *Cerastes vipera* | 1 | 1 | **4.6** | 2.06 |
|  | Disintegrin | A0A2H4Z2Y1 | 9 | *Daboia siamensis* | 1 | 1 | **12** | 2.06 |
|  | | | | | | | | |
| **Nucleotidase**  **(2)** | 5'-nucleotidase (Fragment) | W8EFS0 | 30 | *Macrovipera lebetina* | 12 | 2 | **45** | 27.32 |
|  | Ecto-5'-nucleotidase | T1E3Y5 | 10 | *Crotalus horridus* | 5 | 1 | **64.8** | 10.04 |
|  | | | | | | | | |
| **Nerve Growth Factor (NGF)**  **(4)** | Beta-nerve growth factor | A0A223PK33 | 12 | *Daboia russelii* | 2 | 2 | **27.7** | 5.15 |
|  | Nerve growth factor beta polypeptide (Fragment) | B8QCI0 | 16 | *Daboia russelii* | 2 | 2 | **21.6** | 5.15 |
|  | Venom nerve growth factor 2 | V9I1B5 | 12 | *Daboia russelii* | 2 | 2 | **27.4** | 5.15 |
|  | Venom nerve growth factor 1 | V9I1K1 | 7 | *Daboia russelii* | 1 | 1 | **27.4** | 2.86 |
|  | | | | | | | | |
| **Vascular Endothelial growth Factor (VEGF)**  **(2)** | Snake venom vascular endothelial growth factor toxin VR-1 OS= OX=8707 PE=1 SV=2 | P67861 | 8 | *Daboia russelii* | 4 | 1 | **16.3** | 13.51 |
|  | Snake venom vascular endothelial growth factor toxin VR-1' OS= OX=343250 PE=1 SV=1 | P0DL42 | 10 | *Daboia siamensis* | 4 | 1 | **12.5** | 13.51 |
|  | | | | | | | | |
| **Miscellaneous**  **(5)** | Glutaminyl-peptide cyclotransferases | M9NCG3 | 35 | *Daboia russelii* | 9 | 2 | **42.1** | 14.76 |
|  | 78 kDa glucose-regulated protein | J3RZN4 | 13 | *Crotalus adamanteus* | 6 | 6 | **75.4** | 9.54 |
|  | 78 kDa glucose-regulated protein | J3S4I0 | 13 | *Crotalus adamanteus* | 6 | 6 | **72.1** | 9.54 |
|  | Keratin, type II cytoskeletal cochleal-like | A0A0B8RV71 | 7 | *Crotalus horridus* | 4 | 1 | **53.7** | 9.59 |
|  | Keratin, type II cytoskeletal cochleal-like | J3SCI4 | 7 | *Crotalus adamanteus* | 4 | 1 | **53.6** | 9.59 |

**Supplementary data**


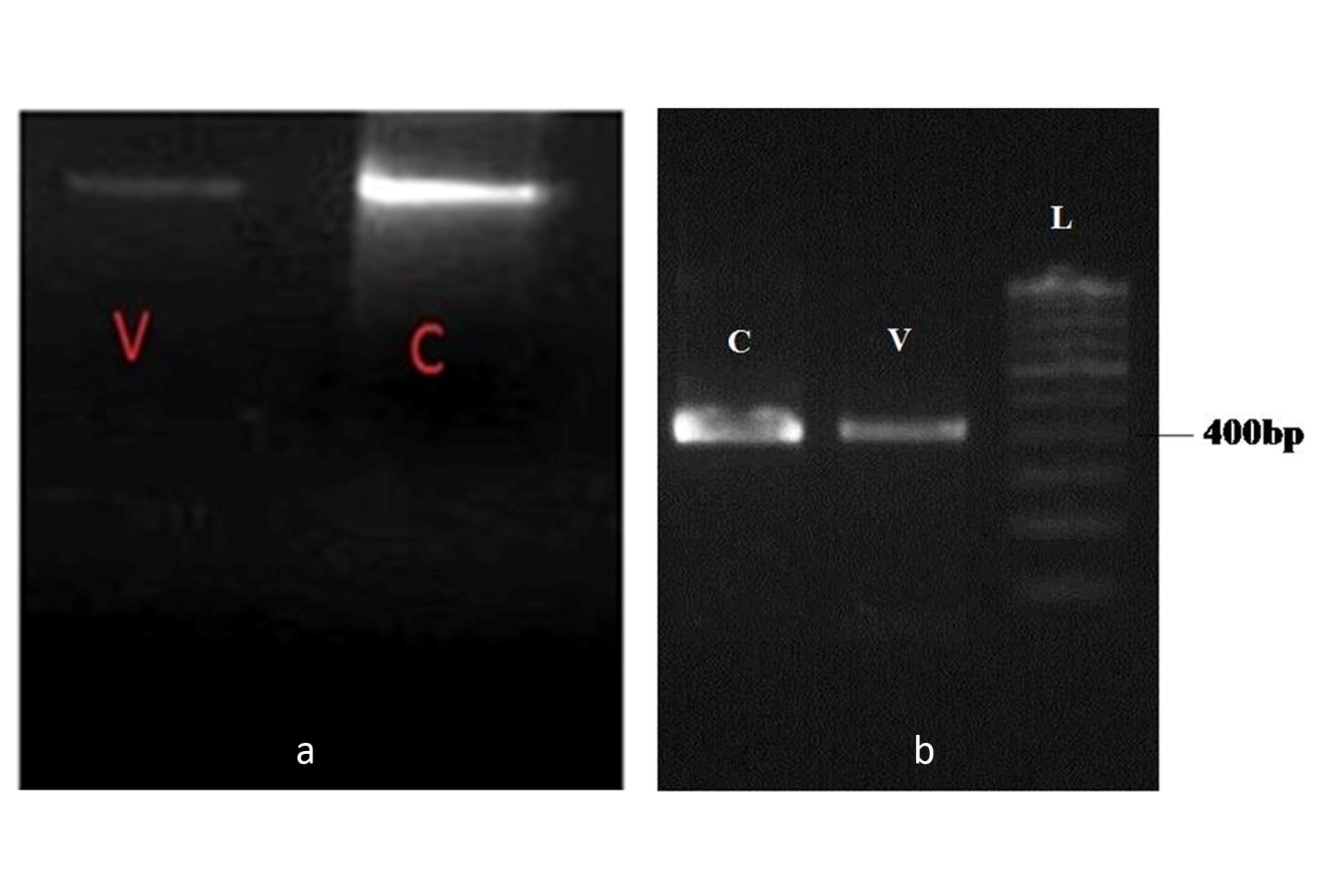


**Supplementary Fig 1:** **(a)** Electropherogram with DNA bands; **(b)** Agarose gel of PCR product of sample. **(C:** Cobra *(Naja naja)* venom; **V:** Viper *(Daboia russelii)* venom; **L:** DNA ladder)

**FASTA FILE**

**Daboia russelii voucher MDU-VV cytochrome b (cytb) gene, partial cds; mitochondrial**

**(**[**https://www.ncbi.nlm.nih.gov/nuccore/MN016938.1?report=GenBank**](https://www.ncbi.nlm.nih.gov/nuccore/MN016938.1?report=GenBank)**)**

GenBank: MN016938.1

>MN016938.1 Daboia russelii voucher MDU-VV cytochrome b (cytb) gene, partial cds; mitochondrial

CCTAACCACCTGACTATGAGGTGGCTTCTCTATCAACGACCCAACACTTACTCGATTTTTTGCTCTCCAC

TTCATCCTACCATTCACTATTATCTCATTATCATCTGTCCACATCCTCCTTCTTCACTATGAAGGCTCCA

ATAACCCCTTAGGAACTAATTCCGACATCGACAAAATCCCATTTCACCCATATCACTCCTATAAAGACTC

CCTGATACTAACAATTCTAATTACAGTCATATTTATAATCCTATCATTTGACCCAAATATAATAAATGAC

CCAGAAAATTTCTCAAAAGCTAACCCCCTAGTCACCCCACAACACATTAAACCCGAATGATACTTCCTAT

TTGCCTATGGAATCTTACGATCAATCCCTAACAAACTAGGAGG

**FASTA FILE**

**Naja naja voucher MDU-CV cytochrome b (cytb) gene, partial cds; mitochondrial**

**(**[**https://www.ncbi.nlm.nih.gov/nuccore/MN006878.1?report=GenBank**](https://www.ncbi.nlm.nih.gov/nuccore/MN006878.1?report=GenBank)**)**

GenBank: MN006878.1

>MN006878.1 Naja naja voucher MDU-CV cytochrome b (cytb) gene, partial cds; mitochondrial

ACCACACTAACAACCTGACTTTGAGGGGGGTTTTCTATTAACGACCCAACCCTCACCCGATTTTTTGCCC

TACATTTTATTCTACCATTTATCATCATCTCACTATCCTCAATCCACATCATCCTACTACACAACGAGGG

CTCTAATAACCCTCTTGGCACCAACTCAGATATCGACAAAATTCCATTCCACCCCTACCACTCCTATAAA

GATGTACTGATAATTACCTCTATGATTACCCTACTACTTCTCATCCTATCATTCTCACCGAGCCTGCTCA

ATGATCCAGAGAATTTCTCCAAAGCTAACCCGTTAATCACACCACAACATATCAAGCCAGAATGGTACTT

TCTCTTTGCATATGGCATCCTC
